# Velpeau’s Clinical Lectnres on White Swellings

**Published:** 1838

**Authors:** 


### REVIEWS

AND

### BIBLIOGRAPHICAL NOTICES.

---

### Art. VI.—White Swellings.

*Memoire extrait des lecons cliniques de M. Velpeau sur les tumeurs blanches; exposition nouville de ces maladies*, par Gustave Jeanselme. (Archives Générales de Médecine.)

Notes of Mr. Velpeau's clinical lectures on White Swellings, with a new view of these diseases—By Gustavus Jeanselme.

This essay embraces the discussion of a very common class of diseases, that are a source of great anxiety and sometimes excruciating pain to the patient, and cause great solicitude to the practitioner, who with mortification, frequently beholds them proceeding to an unfavorable termination despite his untiring and continued efforts.

The very names that have been applied to the disease, are a proof that its pathology has not been well understood. White swelling, scrofulous joints, dropsy in the joints, &c. &c., are terms that have been familiarly used without any very definite import, and applied, without sufficient discrimination, to different diseases, so that, in perusing the works of authors, it is often exceedingly difficult to ascertain the precise meaning they are intended to convey, without a very close comparison and analysis, of the writer's composition.

We find that Mr. Velpeau has introduced a new word, *Arthro-*

*pathia*, which is no doubt etymologically correct, and may perhaps serve better than any other, as a generic appellative for the whole family of diseases of the joints. Still it is no more definite than the others, and is only preferable, because it is so comprehensive that it embraces all forms of the disease from whatever cause they arise, or whatever may be the characters they assume.

We propose to give a brief account of Mr. Velpeau's classification, with a few passing remarks and an occasional allusion to the writings of other authors, and wherever it may be possible, we shall introduce the synonyms, so that the reader may be aided as much as possible by association with familiar descriptions.

Commencing with a classification of the different forms, which appear sufficiently marked by their symptoms, we will present them, from our author, as follows:

"White swellings belong to two great classes of arthropathia: A. of the soft parts: B. of the hard parts; The first class naturally divides itself into three genera; external, internal and capsular; the second is presented under the form of cartilaginous, superficial bony, and deep or parenchymatous bony arthropathia. All of these may be rheumatic, scrofulous, tuburculous, syphilitic, scorbutic, cancerous, &c., or simply inflammatory."

"They may be further noticed as follows:—1st *Extra capsular arthropathia.* Stiffness, sometimes pain; irregular swelling, without effusion into the joint—generally a slight disease; requiring the same treatment as phlegmonous erysipelas, or engorgement of the subcutaneous tissues,—if there be a purulent collection, use the bistoury with boldness."

By *extra-capsular* we suppose our author intends to designate the collections in the Bursæ mucosæ, with which all are familiar. The next form seems to embrace the White swellings of Bell and other authors, and those diseases which Mr. Brodie refers to the ligaments, including also the *Hydarthrus and Hydrops* articuli, which Mr. Brodie attributes to a "diminished action of the absorbents, or to an increased action of the secreting vessels,"—and here we find him at issue with Velpeau, who denies that Bichat or his followers have ever satisfactorily demonstrated a *synovial membrane* on the articulating cartilages—this matter will be further considered as we advance; we now return to Velpeau's:

"2nd. *Pure Capsular Arthropathia*, caused by sprains, external violence, or rheumatic affections; accompanied by pain, on certain movements, and sometimes increased by pressure; causing a swelling of the extra capsular layers; producing internal effusion. It is a more serious lesion than the preceding, and the origin of some that are to be described; It requires active anti-phlogistic medication, and afterwards, resolvent ointments, large blisters, compression; and full doses of mercurials.

3rd. *Specific or blennorrhagic arthropathia*, comes on suddenly; soon characterized by an abundant effusion, with little pain in the first stage, it soon assumes all the characters of an acute arthritis in the second; it requires an energetic treatment, but rather evacuant and revulsive than antiphlogistic; blisters, mercurial frictions, compression, purgation, with large doses of calomel, sometimes also balsamic substances.

4th. *Fungous Arthropathia*. Sometimes primitive, oftener consecutive, always mild and rarely painful; it is announced by elastic, thickened enlargements, sometimes rolling under the finger like a foreign body, there is occasionally effusion, the joint may acquire an enormous volume, without absolute hindrance to progression; it is generally serious from its obstinacy, sometimes resisting every thing, it never yields to blood-letting alone, is improved by the *monstrous blisters*, resolvent ointments, transcurrent cautery, moxas, setons, escharotics, compression and large doses of calomel."

The fungous arthropthia just mentioned appears to bear a very close analogy to the affections described as a "morbid change of structure" by Brodie—and which he considers as owing their origin "to a morbid action in the synovial membrane which loses its natural organization* becoming converted into a thick pulpy substance of a light brown or reddish brown colour, intersected by white membranous lines." He considers the disease peculiar to the synovial membranes, and compares its characters with those of tubercles, schirrous, fungus hæmatodes, &c., in which the natural structure is destroyed and a new one added. He also observes that these changes are most common in the knee joint, which may be attributed to the effect of cold. This form also bears a very great resemblance to the *fungus articuli*, of German writers, and includes a part of what are familiarly known as *loose cartilages*.

The next form is evidently that which is commonly known as dropsy in the joint, and may be very properly referred to an inflammation of the synovial membrane, with consequent effusion.

"5th. *Pure synovial arthropathia*,—Essentially characterized by a serous effusion, without pain or sensible thickening of the articular envelopes, slightly hindering the movements of the joints; requiring purgatives, mercurials, colchicum, or diuretics associated with large blisters and resolvent frictions."

"All these forms have the common character of swelling and super-

*This form also bears a very close resemblance to the *fungus articuli*, of the German writers ficial pain from the beginning, and they never last long without changing the form of the part."

After demonstrating the existence of the synovial membrane, and tracing it over the cartilages of incrustation and interarticular fat, as the conjunctiva covers the cornea, and alluding to its strong functional and anatomical resemblance to the serous membranes, Mr. Brodie says he has never met a case where the disease was proved to have originated in the ligaments, and that no part of the body is much more frequently diseased than the synovial membranes. Cruveillheir, (Dict. de Méd.) gives, as the result of his observations, that in nineteen out of twenty cases, the disease is a chronic inflammation of the tissues, and that even where the other parts are involved, the same proportion will be found to have commenced in the synovial membranes, that they assume a fungous appearance, and that amputation is too often performed; because pus in a joint, is like a cold abscess any where else, with considerable separation and difficult approximation of its walls, and that we have no longer to treat a disease of the ligaments or synovial membranes, but an old abscess which is in an unsuitable state for adhesion.

As Velpeau rejects this view of the pathology; we find that his next is called cartilaginous, it corresponds to what Brodie has described as ulceration of the articular cartilages, and to his affections of the synovial membrane investing them.

"6th. Cartilaginous arthropathia—A mechanical affection, embracing ulceration and contusion of the cartilages, and ulceration of the *synovial membrane*, or what is described as such by Mr. Brodie; resulting from pressure of the cartilaginous surfaces on one another; it may be compared to a crushing, wasting, or excoriation of organic plates; it comes on suddenly, and is announced by a crackling, and by acute pain that ceases entirely during the immobility of the limb, and recurs on certain movements; it is sometimes complicated with bony arthropathia. The cure is at length effected by absolute repose, or after the disappearance of the cartilaginous rugosities."

Of the next two species, which seem to be only varieties of each other, the first does not appear to have been particularly noticed by the writers that we have consulted, though isolated cases occur, in which the lesion appears to have been in the superficies of the bone.

"7th. *Superficial bony Arthropathia.* The cause is generally internal:—announced by dull pain when the joint is at rest, and acute, intolerable pains upon the least motion. The effusion, swelling, &c., are secondary. It is a dangerous lesion; requiring V. S. cups, leeches, mercurials and purgatives, or otherwise, according to the general state of the patient; it is feebly modified by ointments, blisters, or compression; but improved by moxas and cautery, and especially by absolute rest; finally demanding amputation, or terminating in anchylosis."

The following, as the preceding, is supposed to refer to disease in the epiphyses of the bones and not in their shafts, if so, they are paralel to what Brodie calls "Scrofulous disease of the joints, having its origin in the cancellous structure of the bones," and to his "Malignant diseases of the bones."

"8th. *Deep arthropathia of the bones.* It is known by dull and deep pain, during motion or rest, worse at night, accompanied with heat, without swelling at first, and it may last for months or years without effusion; it sometimes invades the cartilages of incrustation, producing an excessively painful affection; it often embraces the head of the bone, when it gives rise to symptoms of mild or acute inflammation, and finally assuming the character of exostosis with inflammation or osteitis; it is always very tedious, frequently requires amputation, and never terminates happily until after the elimination of the necrosed or altered tissues, it especially requires internal remedies; blisters, cautery, moxas, but not compression, nor other topical applications."

"We see that pain is the reigning symptom and primitive sign in the affections of the hard parts, which often exist weeks or even months without any swelling. The class of diseases known under the name of *White swellings* is composed of an infinity of various lesions, and without studying their principal elements it will be impossible to perfect their therapeutics."

We now proceed to introduce a few passages which relate to the treatment of some of the most interesting and comprehensive species of this disease.

*Treatment of Fungous Arthropathia.*—After adverting to the modifying influences of age, sex, and constitution, and making a few remarks on general therapeutics and pathology, our author goes on to say:

"If the patient attribute his malady to some external violence, and is otherwise healthy, we should effect one or more general bleedings and afterwards apply leeches or cups to the articulation, rest must be enjoined, the bath used every two or three days, emollient poultices applied to the part, and the patient should be restricted to vegetable diet and diluent drinks. If the case be advanced general and local bleeding and the antiphlogistic plan should be adopted."

Of other local applications, he recommends the hydriodate of potassa, the ioduret of lead and the mercurial ointment. The former, as a friction morning and evening is considered a useful adjuvant when the disease is in the soft parts, unaccompanied by pain or any symptoms of suppuration, but wholly inadequate and even hurtful in other circumstances than those mentioned. The ioduret of lead is viewed in the same light, but is less irritating and a better discutient. The mercurial ointment enjoys a higher reputation than either, with Mr. Velpeau, who recommends its use, after rigid antiphlogistic treatment according to the directions laid down by Bell; it must be long continued, associated with bathing, and its effect upon the gums must be watched.—We now come to the favorite remedy of Mr. Velpeau and we will quote his remarks upon the subject.

"In the neighborhood, or at a distance from the part, blisters are of doubtful utility, but placed upon the very joint they have effected cures. I have seen great benefit derived from the usual mode of applying a number of small blisters around the joint, but innumerable trials have convinced me that the blister may be infinitely more efficacious in another form. I direct it so large as to envelope the whole joint and extend an inch beyond the swelling. In this way there is no more pain than usual, nor is its effect upon the urinary organs much increased, and it may be moderated by the use of camphor. Febrile reaction is not to be dreaded, and the change sometimes effected is truly remarkable. I have used it more than two hundred times in five years, and can affirm that it never appeared to aggravate the disease, whilst it has been generally impossible to doubt its efficacy. We may always calculate upon its affording relief when the affection is confined to the parts between the capsule and integument; chronic phlegmasiæ of the capsule yield rapidly to it, and even obstinate fungous arthropathia is powerfully controlled by this agent, when the hard parts are not involved; but sero-synovial effusion is conquered in a truly astonishing manner by these *monstrous blisters.*"

" Mr. Velpeau urges practitioners to make a trial of the bath first, the next day the blister is applied and left on twenty-four hours, if the tumour is not very irritable, the cuticle is immediately detached; otherwise the serosity is drawn off by clipping the vesicles. The denuded surface should be covered with paper spread with cerate, to be renewed every morning; this dries up the suppurating surface in a week or less. The patient is then left quiet as long as the tumour appears to diminish, when a second bath and blister are prescribed. The first effect of this powerful topical treatment is to soften the tumour and render it more fluctuating, which is often observed from the second day. Sometimes the volume of the tumour diminishes almost immediately; but oftener remains stationary, or even seems to increase for a day or two, and the capsule does not empty itself until the blister is desiccated."

Some cases are cited which go to strengthen this testimony; Mr. Velpeau recommends that other means which may be indicated should be associated with the blister.

Mr. Velpeau seems to set a lower estimate upon escharotics and moxas, though he recommends them in deep-seated arthropathia; in general, he thinks they should not be used until after an unsuccessful trial of other modes of treatment, and that they are rather adapted to complete a cure already in progress, than to commence with.

He very properly denounces the use of setons passed through the capsule, and considers their application elsewhere, as deserving no more confidence than an escharotic or moxa.

*Compression.*—Mr. Velpeau considers this agent too much neglected by Surgeons, having witnessed its great efficacy in the hands of Brétonneau at Tours, he determined to introduce it into the hospitals at Paris. He complains of the ridiculous lengths that some of its partizans went in extolling it, and of their designating even the different degrees of compression to be exercised, he says:

"Compression is best effected by a roller bandage, care being taken to fill up all cavities or depressions about the joint, with graduated compresses, and the whole so disposed that equal pressure may be made upon all parts; it should begin below the joint and extend a few inches beyond the diseased part. At first moderate, it may be afterwards increased according to the degree of irritation it appears to produce. It does not interfere with the use of ointments, and I have frequently combined it with blisters. When the joint has nearly resumed its normal dimensions, and we fear to relax the compression, lest the tumour return, it may be rendered permanent by pasting each surface of the bandage with starch, and applying bits of pasteboard.* The whole apparatus is moulded exactly to the shape of the tumour, and when the bandage dries, the patient may rise and walk about without apprehension, for the articulation is necessarily immovable, and it is almost impossible for it to swell again. Strips of adhesive plaster, applied as for ulcers of the legs, act mechanically and *chemically*. I have used them with every appearance of real benefit, but this plan is expensive, and requires more time and care in the application."

*The Actual Cautery*, has been much vaunted by many writers, but now scarcely used, because of the great terror it generally inspires, and the consequent objections of the patients. Mr. Velpeau considers it highly beneficial in some forms, particularly where there exists sero-synovial effusion. He particularly recommends the *transcurrent cauterisation*,† which he has seen bring a-

* Mr. Velpeau has for some time treated all fractures of the lower extremities in this way.

† This consists of a cautery two inches long, applied in the great lateral diameter of the joint with smaller strokes on each side, like the branches of a fern froud.

bout the resolution of engorgements and effusions that had resisted all other means, and says it effects a radical cure.

The *Douche* is considered an accessary which should not be neglected, but possesses no great power. Among the internal remedies, emetics and purgatives have not furnished any encouraging results—diluent drinks are recommended. The mercurial preparations so much extolled in England, appear not so highly valued in France; attention has recently been aroused by the publications of O'Bierne; but Velpeau thinks that he was mistaken in the diagnosis of the cases he reports, in which calomel was so successful, still, he admires the energetic course pursued, and says he has adopted a similar plan for some years—his prescription is as follows:

℞ Calomel, - - - grs. vj. to x.
Ipecac. - - - - grs. xij. to xx.
Rhubarb, - - - grs. xv. to xxx.

Mixed and divided into four papers. S. to be taken during the 24 hours.

We will quote the results of his practice on the plan of Mr. Brodie, giving from ten to twenty-four grains of Calomel per day, associating with it, from one to four grains of opium.

"In Arthropathia without alteration of the hard parts, and without fungous change of the capsule, the patient has improved rapidly, and the joint has been almost completely reduced in one or two weeks. When the capsule was much thickened, externally or internally, most patients were improved by the treatment, but the melioration was soon checked, and it was necessary to resort to other means to complete the cure. In recent cases with pain or other inflammatory symptoms, large doses of calomel pushed to salivation, generally exerted a happy influence in modifying the disease. In other cases, the mercurial treatment produced uncertain, and sometimes hurtful results, so that I now use calomel only, in recent cases with sero-synovial effusion, when local or general bleeding is inadmissable, and in old cases of the *hydarthrose* form; and generally associate it with external means when there is engorgement, thickening and alteration of the capsule, giving from ten to twelve grains the first day, with two of opium, and fifteen to eighteen the next day, if not contra indicated; whether there be ptyalism or not, the remedy is suspended about the sixth day, to be resumed in a week."

"Preparations of Baryta: were much praised during the last century by Crawford and others, and it has lately become a sort of panacea. In Germany and Italy the muriate has deen used in large doses, on the contra-stimulant method, but is now generally renounced. According to the plan of Rasori and Pirondi, I gave it in doses of four to six grs., in four ounces of distilled water, for four or five days, then twelve, fifteen and twenty grains, and even forty grains in twenty-four hours.—

Many patients took it for one or two months; some soon experienced nausea, colic, and diarrhœa, so that it was necessary to suspend it; others felt no inconvenience from its use. I must say that negative results alone have been obtained. The success attributed to it, is no doubt due to the associated vegetable diet, to the early stage, and slight form of the cases submitted; it engages the attention of the patient, and we gain time, and since time and rest are the chief elements of treatment, it may impose upon superficial observers. I have not used the preparations of iodine internally to any extent, but fear their action."

We now come to the consideration of *Arthropathia of the hard parts*, which, in accordance to the pathological views of Mr. Velpeau, embraces what is generally considered inflammation and ulceration of the synovial membrane investing the cartilages, affections of the cartilages themselves and those curious morbid growths that have been styled "loose cartilages." These are very ingeniously accounted for by our lecturer, but we do not find that he adverts to an operation for their removal, which has been successfully performed by British Surgeons, and also in this country, by a fellow citizen of ours, who operated in New-York with complete success, though he admits with others, that it is scarcely feasible, or at least, that it is attended with eminent risk.

We have heretofore alluded to Velpeau's views and expressed surprise at finding him differing from so many high authorities.—We will quote a few paragraphs entire, as they relate to very interesting forms of the malady we have been considering, and contain an exposition of his views upon the pathological anatomy of the parts concerned.

"*Cartilaginous Arthropathia.* In accordance with the ideas of Bichat, nearly all writers have described diseases, belonging exclusively to a pretended synovial membrane of the cartilages themselves, as if it had any thing to do with the other organic tissues of the body. Thus we see in most authors, that this disease of the joints is attributed to an inflammation or thickening, by ulcerations or some other organic and vital lesion of the synovial membrane of the cartilages. I cannot agree with the generality of Surgeons in this opinion;—first there is no synovial membrane on the surface of the cartilages—moreover, I do not fear to affirm, that the incrusting cartilages are insusceptible, primitively, of any sort of inflammation, thickening or ulceration. As they are really, mere crusts, without vascularity—they can only be secondarily affected, or, as inorganic bodies, like the hair, nails, epidermis, or the enamel of the teeth—that is, only mechanically and chemically. Still it would be wrong to deny the disease described by Mr. Brodie, who is only mistaken as to the situation of the affection he mentions."

"The presence of vegetations or fungi that have been observed on the surface of the cartilages, is explained in the following manner:—1st. They either depend upon fungous plates originally deposited upon the surface of the bones, and which being successively detached and raised, destroy the cartilage, making it appear embraced in the change; or 2nd, that these fungi, were only the result of an effusion of plastic organized lymph on the synovial surface of the bones, and afterward prolonged even into the interior of the joint. In these two cases it is possible to find, on a close examination, cartilaginous plates that are thinned, eroded, and moveable, as foreign bodies in the midst of the vegetations. Besides, plates are observed, still adhering to the subjacent bone, as in the normal state, with their free edges *thinned.* In my clinique, I have shown multiplied cases of diseased joints in full suppuration for months and even years; but you have seen that in these articulations the cartilages did not present any of the alterations generally attributed to them. I have frequently seen, and you may have observed, in the patients under care, the cartilaginous heads of the bones that were laid bare by accidental wounds, or by disarticulation, insensibly disappear by falling off, by wasting or by dissolution, but they never became vascular nor thickened otherwise than by imbibition. I repeat then in a familiar manner, what I have elsewhere exposed, in my *Traite* d' Anatomie chirurgicale, and under the word Articulation, in the Dictionary of Medicine, 2nd edition; that the cartilages of incrustation, and what has been designated by the name of synovial membrane of the cartilaginous surfaces, are insusceptible of any *primitive* organic disease."

"We still find an arthropathia arising from a wasting or some mechanical alteration of the articulating cartilages, particularly in old men and those that walk a great deal, the joints are dry or shrivelled, the cartilages are wasted by friction, and become thin, rough, and sometimes so unequal, as to be furrowed with grooves. In proportion to the depth of the affection, it produces only a slight dryness, pain and crepitation in the joint: but in subjects of middle age, with similar alterations, the jarring occasioned by the motions of the limb, are soon transmitted to the neighboring bony surfaces, so as to irritate them, to produce pain, and to cause another kind of arthropathia which will be soon described."

"The cartilages are liable to be contused or crushed. It may be easily conceived that falls or external violence so directed as to bring the ends of the bones in close contact and cause violent pressure between them, may contuse or even grind the incrusting cartilages more or less extensively—again, pressure obliquely directed, may detach or break off plates which will be isolated within the articulation. We can easily understand how such alteration of the cartilages would disturb all the motions of the joint, and give rise to most of the symptoms which would arise from the presence of a foreign body."

"This variety may be known by the following characters:—If the patient be not of advanced age, some forced march, fall, misstep or other external violence will be found the primary cause of the evil. Pain will have been felt before observing the least swelling—this will be perceived in certain movements, and disappear in other positions; sometimes it will be so excessive as to cause the patient to fall, and will occasionally bring on syncope. As the pressure of the soft parts does not increase the pain, it will cease entirely with rest; and may be again produced by putting the limb in such position as to create friction. The pain will be more or less acute in proportion to the depth of the roughness on the cartilages, and to the extent of the injury toward the bone."

"The prognosis is worse here than in the affections of the soft parts, but better than when the bones are affected. It is serious because the injury of the cartilages is irreparable, but less serious than when the bones are affected, because, the affection being purely mechanical, the general organism does not receive so deep a shock. We rarely obtain a complete cure, but frequently some kind of anchylosis."

"The treatment differs from that already mentioned—general bleeding, mercurial and iodine ointments are rarely indicated; compression is at first of no avail. The treatment must be long continued; that which succeeds best, consists at first, of complete immobility, and it is very necessary to keep the limb extended in well furnished splints, or in a starched apparatus, with little blisters,moxas, escharotics, actual cautery, used as revulsives or derivatives. Internal medicines, whether preparations of iodine, colchicum, blisters, or the mercurials, are equally indicated—in fine, arthropathia of the cartilages requires; 1st, immobility of the joint;—2nd, some emissions of blood, if the circulation require it;—3rd, the trial of calomel or wine of colchicum in large doses for one or two weeks;—4th, the muriate of baryta for a month—and afterwards by way of topical applications, blisters, moxas, cautery, setons, or the transcurrent cautery. To all this there must be added an appropriate regimen, baths or douches."

"*Arthropathia of the bones.* We find in the articulating heads of the bones, all the diseases that are elsewhere observed—among them, caries and necrosis, fibrous, schirrous, encephaloid, tubercular, and hydatid, growths. If the arthropathia begin on the surface of the bone it may offer two types which we must not confound—either the region covered with cartilages is primarily affected, or the disease first affects the circumference of the head, not thus covered but within the articulation. Whether it be caries or any other lesion, the symptoms are nearly the same."

"Cartilaginous arthropathia of which I have already spoken, often runs into arthropathia of the bones, though the latter frequently exists without the former. The surface of the bone becomes vascular and softens, the cartilage is detached or becomes thin, the bony surface is gradually covered with vegetations and fungous appearances which occupy one or more points or the whole, and which resemble the growths of a bad ulcer. Beneath them are found the carious, necrosed, tuberculous, or cancerous points, or a simple osteitis."

"The disease sometimes arises from an external cause, sometimes from an internal or hidden origin. From the beginning, dull, deep seated pains are felt, which become acute on certain movements, but which persist during perfect repose. The swelling is not early developed and when it comes on, it is by an effusion of liquid into the joint, rather than by thickening of the soft parts. When the disease is more advanced, the interior of the articulation becomes so sensitive that the least jar or motion causes the patient to cry out, and the slightest touch of the covering, or flexion of the limb, produces great pain. Thus we see patients devote their whole attention to preserving immobility of the affected joint; the uncontrolable spasmodic action of the muscles, and the unequal weight of the bones which renders it impossible to avoid pressure on the articulating surfaces, are especially tormenting. This applies only to the arthropathia of the bones corresponding to the cartilages; beyond these plates indeed, the disease is nearly similar to that which occurs in other parts. It is the internal and external face of the periosteum which covers the capsule, that becomes affected, or a portion of the bone covered by this part of the fibrous envelope. Here the pathological alterations may be the same, but as the altered part is not subject to pressure by supporting the bones in their movements, the symptoms are not so severe. The pain will appear less deeply seated, it will not be much increased by pressure around the joint, and slightly increased by motion; there will be less spasmodic action of the muscles, and the sleep and repose of the patients will be much less disturbed."

"In all these cases, if the disease have lasted a long while, and if it have produced pus, the cartilages may often disappear by dissolution, without there being any fungous growths on the bones, and even so that the articular heads of the bones may sometimes appear smooth as if burnished."

"On the contrary, whenever the malady commences in the parenchyma of the bones, it first exists in the form of simple osteitis, caries, necrosis, &c. When developed in a particular constitution, or by any specific cause, or when it arises from an internal origin, or depends upon any external cause whatever, it may be easily distinguished from the forms already described. The patients are warned of its approach by a dull, deep, intermitting pain, generally worse at night, often more decided during rest than motion. The part becomes *hard*, and weak; but the movements of the joint remain free, and there is no trace of effusion into the articulation. If swelling come on it is not easily observed. Perhaps, after all, this variety is rather a disease of the bones than an arthropathia, for the disease from the beginning may be foreign to the joint."

"After awhile the arthropathia of the parenchyma will offer symptoms still more easily observed; they will differ according to the mode in which the affection is propagated: thus, whether it arise from caries or necrosis, &c., or from suppuration of the bones, tubercles, cancer, &c., the disease may progress towards the cartilage, or in an opposite direction, or again towards the circumference of the extremity of the bone. In the first case, the symptoms of superficial arthropathia of the bones will come on, the cartilage will be loosened, the pain will become excessively acute effusion will be established into the joint and we shall have all the symptoms above described. In the second case, the pain will remain dull, it may become lancinating, though still deep, it will increase towards the middle of the limb; and how acute soever, it will not hinder exercise of the joint. It will generally be a long time, before swelling and irregular lumps, announce exostosis and decide the true nature and precise seat of the disease. When, instead of being propagated in the axis of the bone, the disease proceeds to the circumference it points under the periosteum which is in contact with the synovial capsule, and then it becomes completely a superficial arthropathia of the bone; sometimes it reaches the circumference without the limits of the febro-synovial capsule, and then we are advised of it by fixed, hard, painful lumps, situated on the circumference of the diseased bone, while the joint remains free in its motions and without effusion. The pain is increased by pressure on some one part, while no pain is felt in the neighborhood."

"The prognosis is always serious: though varied according to the form of the disease. Indeed, tubercular, cancerous, and scorbutic affections are always more serious, near the joints than simple caries and its consequents. But this gravity belongs in such cases to the nature of the affection and not to its seat; *superficial* bony arthropathia, is *cæteris paribus*, more grave than *deep* affections of the joints, and when the former corresponds to the cartilages of incrustation, it is much worse than at their periphery. It will be seen, that the first, almost necessarily brings on the loosening and destruction of the cartilages, that suppuration is almost inevitable, and that the functions of the limb must be forever lost, if indeed, amputation be not imperiously demanded. But when the periphery is affected, the periosteum or the fibro-synovial membrane alone will be altered, and as the articulating surfaces remain sound, the disease may last a long while, or at length disappear and the joint preserve its natural functions."

"Deep seated arthropathia of the bones is much more serious when it affects the cartilages; it then assumes at once all the dangers of the superficial form, and when it produces thickened masses of altered tissues, a cure appears scarcely possible, unless the altered part be eliminated by inflammation or removed by an operation. If, on the contrary, the disease follow another direction, the patient may continue twenty years without any further inconvenience than fistulous openings, and an occasional abscess arising from exostosis with inflammation and suppuration, until the irritation is thrown off—when the ulcer will be modified and healed."

"The therapeutics of this disease are still quite incomplete. As we are often influenced by the general state of the patient, and by his other maladies, the strictest attention to hygeine and general medication is necessary. In superficial arthropathia of the bones, large doses of calomel are useful, but they must be suspended for a fortnight, so soon as salivation is threatened; but when the disease is deep seated this medicine seems to be hurtful. The preparations of Barytes do not seem to deserve the least confidence in these cases, The same may be said of the iodurets, they should be used only to gain time."

"All forms of this disease in the bones, may be followed by purulent collections in the articulations, which occasion reaction, diarrhœa, burning heat, irregular chills, in fine, a sort of purulent fever. If the whole joint be thus affected the only remedy to propose, is amputation of the limb. But these foci are sometimes found in isolated points, or in the shape of nodes on the periphery of the joint, the first idea then, is to give the pus vent; but the opening of such an abscess is a grave question. Experience proves indeed, that opening an abscess that communicates with the cavity of a large joint, is likely to produce all the symptoms of an acute purulent arthropathia, and consequently to endanger the life of the patient. If the pus arise from an alteration of the shank of the bone without connection with the joint, the opening of it is unattended with danger."

We have now followed Mr. Velpeau through some of his nice distinctions of diagnosis, and observed his bold and energetic treatment, and hope that benefit may be derived from an application of some of the principles inculcated; though we must still express our partiality to Brodie whose views of the pathology appear more in accordance with sound deductions and analogy.

